# Stroboscope Based Synchronization of Full Frame CCD Sensors

**DOI:** 10.3390/s17040799

**Published:** 2017-04-07

**Authors:** Liang Shen, Xiaobing Feng, Yuan Zhang, Min Shi, Dengming Zhu, Zhaoqi Wang

**Affiliations:** 1 Virtual Reality Laboratory, Institute of Computing Technology, Chinese Academy of Sciences, Beijing 100190, China; fengxiaobing@ict.ac.cn (X.F.); zhangyuan@ict.ac.cn (Y.Z.); mdzhu@ict.ac.cn (D.Z.); zqwang@ict.ac.cn (Z.W.); 2 School of Computer and Control Engineering, University of Chinese Academy of Sciences, Beijing 100049, China; 3 School of Control and Computer Engineering, North China Electric Power University, Beijing 102206, China; shimin01@ict.ac.cn

**Keywords:** charge-coupled device (CCD), smear, stroboscope, video capture, synchronization, full frame, hidden Markov model (HMM)

## Abstract

The key obstacle to the use of consumer cameras in computer vision and computer graphics applications is the lack of synchronization hardware. We present a stroboscope based synchronization approach for the charge-coupled device (CCD) consumer cameras. The synchronization is realized by first aligning the frames from different video sequences based on the smear dots of the stroboscope, and then matching the sequences using a hidden Markov model. Compared with current synchronized capture equipment, the proposed approach greatly reduces the cost by using inexpensive CCD cameras and one stroboscope. The results show that our method could reach a high accuracy much better than the frame-level synchronization of traditional software methods.

## 1. Introduction

In the past few decades, image sensors have been widely used in industry and daily life. The rapid development of image sensors has also received increasing attention in computer graphics and computer vision research. Image based or video based approaches have been developed for the reconstruction of opaque objects [1,2], flames [3,4,5,6], gases [7], water surface [8,9], mixing fluid [10], humans [11], etc. Information extracted from these approaches is valuable for a variety of applications, such as re-rendering the objects, developing data-driven models and improving results for physically-based simulation methods [3,12]. In addition, the image sensors are also used to track [13] and size particles [14].

CCD (charge-coupled device) [15] and CMOS (complementary metal oxide semiconductor) [16] are two basic types of camera sensors. CMOS sensors have been associated with energy efficiency and fast data-throughput speed, while they would suffer more visual noise and distortion compared with CCD sensors. CCD chips theoretically provide better quality images, but they will produce undesired bright spots or lines when shooting bright objects, such as the sun. This kind of effect for CCD sensors is called smear. Specifically, there are three types of CCD chips: interline transfer, frame transfer and full frame CCD [17]. In interline transfer CCD sensors, every pixel has a charge storage area next to it, so that the charges from the explosion period could be quickly shifted to the storage pixel area that facilitates faster frame rates. Since the pixel storage areas, which transport the pixel charges to the final image, are masked so that light cannot hit them, and the interline transfer CCD sensors could minimize image smears. However, the storage area occupies half of the whole pixel area, which would reduce the area of each pixel available to collect light. Therefore, the interline transfer has a relatively lower Fill Factor (the ratio of a pixel’s light sensitive area to its total area) and is less sensitive. In terms of the frame transfer CCD sensors, they have a duplicate sensor used for storage below the active sensor, so they do not share active pixel area with the storage pixel area and have 100% Fill Factor. However, frame transfer CCDs would suffer badly from smear, which does the same as full frame CCD sensors. Unlike the interline and frame transfer CCD sensors, the full frame CCD sensors have no pixel storage area, which makes the sensor less expensive. In addition, the full frame CCDs have 100% Fill Factor, so they are widely used in inexpensive consumer cameras. Therefore, in this paper, we focus on the full frame CCD sensors.

Smear lowers the quality of images generated by CCD sensors. Several approaches are proposed to remove or reduce the effect of the smear, such as the optical black region detection method [18], the wavelet transform based approach [19] and the image post-processing algorithm [20]. Rather than de-smearing, this paper tries to present a synchronization approach for the full frame CCD sensors by utilizing the smear effect.

Traditionally, industry cameras are used in scientific research due to their high-accuracy synchronization by the inherent hardware. These cameras are expensive, costing at least 700 US dollars per camera and the high prices limit their broad applications. To use consumer cameras in research, the key obstacle is the synchronization problem, due to the lack of synchronization hardware. Several software methods have been proposed to overcome the obstacle. Previous approaches on the synchronization of multiple video sequences are based on feature tracking and geometric constraints [21,22,23]. Unfortunately, some phenomena, such as flames and smoke, contain no obvious features to be tracked from their videos. A different method, based on detecting flashes, has been presented by Shrestha et al. [24], and frame-level synchronization can be achieved through this work. To solve the rolling shutter shear and the synchronization problem of CMOS consumer-grade camcorders, Bradley et al. [25] proposed two methods: the strobe illumination based method and the subframe warp method. However, phenomena like flames and explosions change rapidly and irregularly; therefore, the synchronization accuracy of frame-level or the subframe warp [25] is unacceptable for capturing these phenomena simultaneously. Casio (Tokyo, Japan) designed a consumer camera that could work with synchronization to other cameras [26]. However, the synchronization only works for the Casio EX-100Pro cameras and the number of synchronized cameras is up to seven. In addition, the price is about 800 US dollars per camera, which is even more expensive than some industrial cameras.

In this paper, we present a stroboscope based synchronization method for full frame consumer CCD cameras, which can cost as low as 100 US dollars per camera. In brief, the synchronization is realized by two steps:

- Aligning the frames from different video sequences. The smear dots of the stroboscope are used as the time stamps, and the relative position between the stroboscope and the smear dots in images are adjusted to align the frames from different sequences.
- Matching the sequences. The stroboscope is utilized to generate periodic flashes, which indicate the overlapping content and allow for determining the offset time between cameras. The sequences are matched by matching the flashes using a hidden Markov model.

## 2. Materials and Methods

In this section, we first briefly review the architecture of the full frame CCD. Then, we describe the generation of the smear effect, followed by the analysis of the smear dot generated by shooting a stroboscope. Finally, we show the details of the frame alignment and sequence matching method. The consumer CCD cameras used in this paper are ten Canon PowerShot G12 cameras (Tokyo, Japan), supporting to capture videos with 1280×720 resolution at 23.976 frames per second (fps) frame rate. In terms of the stroboscope, we use a Monarch Instrument Nova-Strobe dbx (Amherst, NH, USA). The dbx has flash rates ranging from 0.50 to 333.33 flashes per second that are adjustable in 0.01 step increments.

### 2.1. Full Frame CCD Architecture

**Figure 1:**
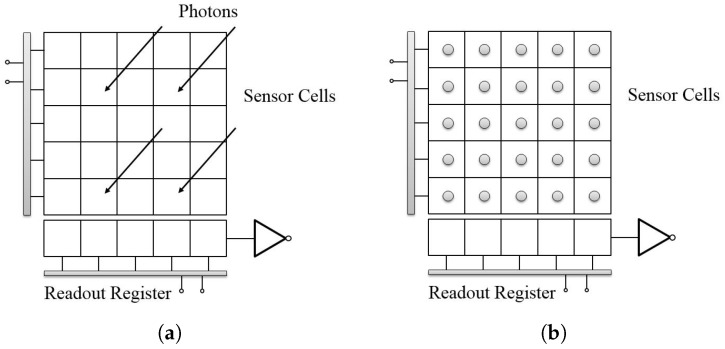
CCD (charge-coupled device) acquisition phase. (**a**) shows that the incoming photons fall on the sensor cells; and (**b**) shows that the photons are converted to electrical charges.

**Figure 2:**
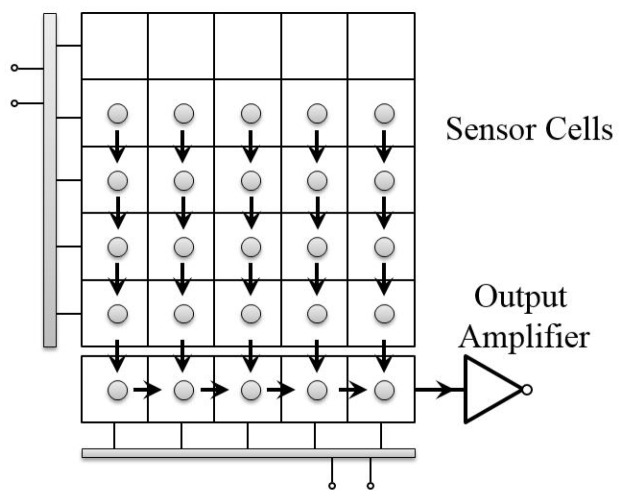
CCD readout phase. Charges are handled row by row to generate the final image through the vertical transfer, horizontal transfer, voltage conversion and amplification processes.

For the full frame CCD sensor, the whole imaging process could be simply divided into two phases: the acquisition phase and the readout phase. In the acquisition phase, incoming photons fall on the full light sensitive sensor cells, and then the cells convert the gathered photon to electrical charges, as shown in Figure 1. In the readout phase, shown in Figure 2, the charges are vertically transferred to the horizontal readout register row-by-row. For each row, after the horizontal transfer process, the charges are then converted to the voltage information, and, finally, the digital data for the image are achieved through the amplifier. The final image is generated by the same operations for all rows of the cells in the sensor.

The frame rate is a common feature for a video camera, and the inverse of the frame rate is the time, here denoted by $t_{period}$, needed for the CCD to acquire an image and read the image out. Hence, the period could be modeled as:

(1) $$t_{period}=t_{acq}+t_{read},$$

where $t_{acq}$ denotes the time for acquiring an image, mainly occupied by the exposure process. $t_{read}$ denotes the time for reading out an image as we described above. In detail, $t_{read}$ can be presented as:

(2) $$t_{read}=t_{image}+t_{mis},$$

where $t_{image}$ denotes the time cost by the transfer of the pixels for the final image. For CCD cameras, there are always extra rows in the sensors besides the rows for the final image. The time needed for transferring the extra rows and other miscellaneous work is denoted by $t_{mis}$. Assuming the resolution of the images is m×n, we get:

(3) $$t_{image}=nt_{perrow},$$

where $t_{perrow}$ denotes the time needed to transfer one row of the image, which could be used to evaluate synchronization error.

### 2.2. CCD Smear

**Figure 3:**
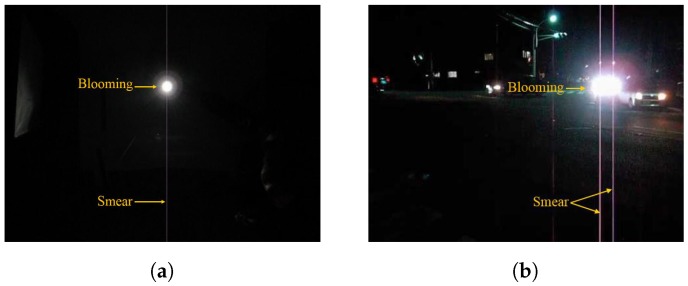
CCD blooming and smear. (**a**) A CCD captured image with blooming and smear, (**b**) another scene image with blooming and smear. Blooming denotes undesired bright sections surrounding the bright light source, caused by charges leaking from one pixel into adjacent pixels. Smear denotes the undesired bright sections above and below the bright light source, caused by charges’ accumulation of the light source during the vertical transfer process.

When there are very bright spots in the scene, blooming and smear effects would appear in the images for CCD sensors, as shown in Figure 3. Blooming is an effect where the charge accumulated on a pixel leaks into adjacent pixels and corrupts the scene [27]. It diminishes the accuracy of the pixel data as information from one pixel is then presented in adjacent pixels. Another undesired effect for the CCD sensor is the smear. If an intense light source is imaged onto the CCD image sensor, undesired signals appear as a brighter vertical (from top to bottom) stripe emanating from the light source part of the image. The undesired brighter sections are called “smear”.

**Figure 4:**
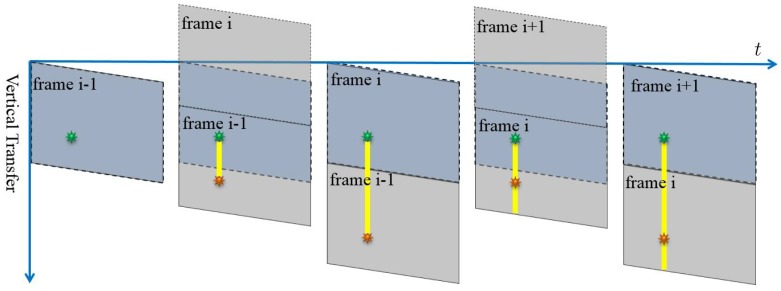
The process of the smear generation of a light source with constant lighting for frame i. The dark blue area indicates that the image sensor area and the gray area indicates the generated image. The orange sun symbol stands for the light source position in the final image and the green sun symbol stands for the light source position of the image sensor. The yellow line denotes the smear.

Smear is produced by the incident light accumulation in the vertical transfer process. While the charges are transferred to the readout register, the sensor cells still accumulate lots of photons from the light source, which leads to undesired vertical bright stripe in the final image. Figure 4 illustrates the whole process of the smear generation by shooting a light source with constant lighting.

### 2.3. Smear of a Stroboscope

**Figure 5:**
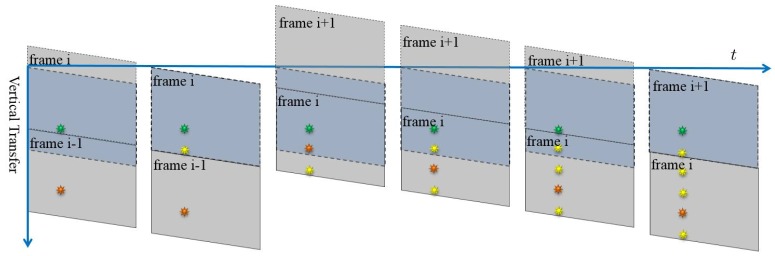
The process of the smear generation of a strobe light source for frame i. The yellow sun symbol denotes the smear. In the whole timeline to generate frame i, only the moments when the strobe turns on are shown.

**Figure 6:**
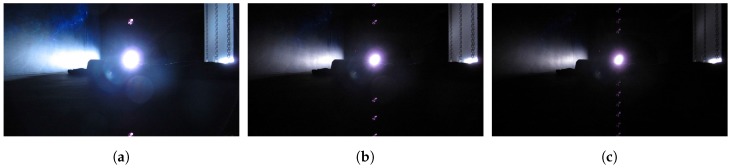
CCD smear dots. The video frame rate is 23.976 fps and the rates of the stroboscope are set as 47.95 (**a**); 191.81 (**b**); and 333.33 (**c**) flashes per second separately.

**Figure 7:**
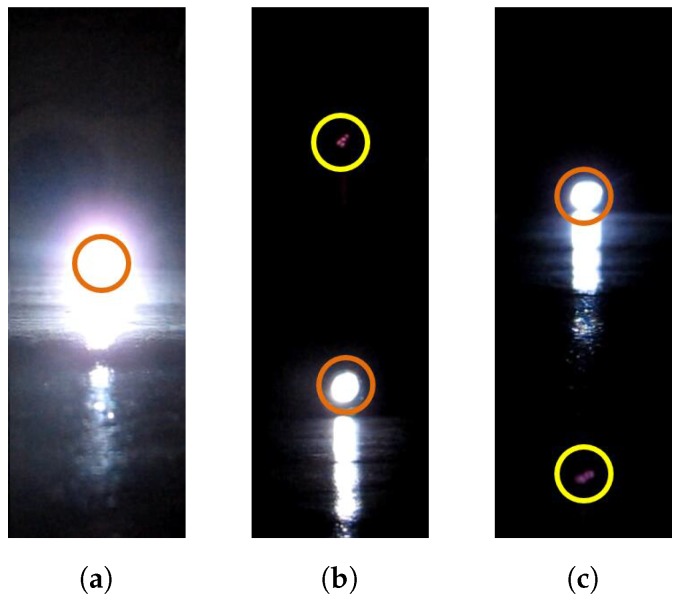
Smear effects when the video frame rate equals the flash rate of the strobe light. The orange circle indicates the light source position and the yellow circle indicates the smear dot position. (**a**) shows that the strobe turns on in the acquisition phase; and (**b**,**c**) show the situations in which smear dots appear above and below the strobe position separately.

If the light source is changed to a stroboscope, the smear would appear as several dots instead of straight lines. The smear dots generation process is illustrated in Figure 5. Only the moments when the strobe is turned on are shown; in other words, the strobe is off in other moments of the timeline for generating the frame i. When the flash rate is set to different numbers, there would be a different numbers of smear dots in the final images, as shown in Figure 6 and Figure 7.

From Figure 6, we could see that when we set a much higher flash rate than the CCD frame rate, there will be several bright dots (smear) in one image. We can use two adjacent dots to compute $t_{perrow}$ as:

(4) $$t_{perrow}=\frac{1}{\Delta d_{smear}f_{flash}},$$

where $\Delta d_{smear}$ denotes the distance in rows between two adjacent bright dots on the same side (up/down) of the light source in the image, and $f_{flash}$ is the flash rate which we can get from the strobe instrument.

As shown in Figure 7, when the flash rate equals the video frame rate, we see that the number of bright dots may be zero (the strobe turns on in the image acquisition phase, shown in Figure 7a) or one (the strobe turns on in the image readout phase). In the one bright dot case, the dot could be either above (Figure 7b) or below (Figure 7c) the strobe light.

### 2.4. Frame Alignment

**Figure 8:**
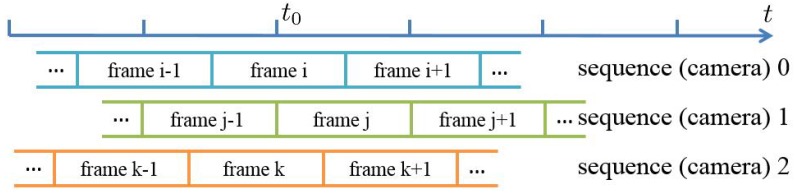
Frames without alignment. The cameras fail to simultaneously capture an event at time $t_{0}$ because frames from different sequences start to record at different times.

When the video sequences of different cameras are captured randomly, the frames from different sequences may not be well aligned, as shown in Figure 8. All three of the cameras aim to simultaneously capture an event at time $t_{0}$. Since the frames are not well aligned, the corresponding frames may not start to record the event from the same time. For example, frame j of sequence 1 starts to record the event at the beginning of the frame (in the acquisition phase), while frame k of sequence 2 may miss the event because the event happens in the readout phase of frame k. Therefore, to synchronize the video sequences, we should first align the frames from different cameras.

**Figure 9:**
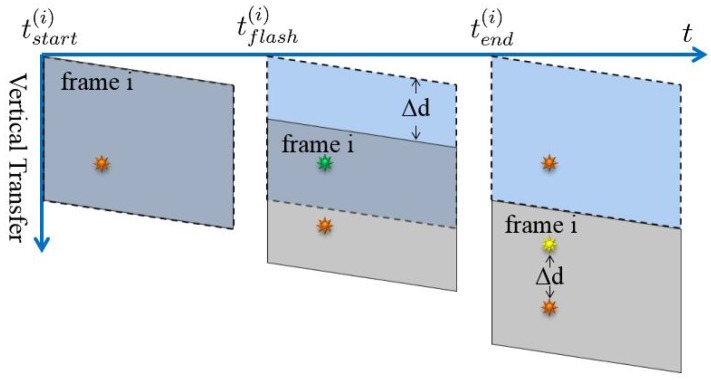
The generation process of the smear dot above the light source position. The orange sun symbol denotes the position of the light source in the image, and the yellow one represents the smear. When the strobe illuminates, the light source turns green.

As shown in Figure 7, when the flash rate of the stroboscope is set equal to the video frame rate, there would be only one smear dot above (Figure 7b) or below (Figure 7c) the actual strobe position in the image. Figure 9 illustrates the generation process of the smear dot above the strobe position.

If the smear dot is above the strobe position, the smear is generated during the readout phase of the current frame, shown in Figure 9. The distance in row ( $\Delta d^{\left(i\right)}$) between the bright dot and the light source for frame i can be expressed as:

(5) $$\Delta d^{\left(i\right)}=\frac{t_{flash}^{\left(i\right)}-t_{start}^{\left(i\right)}}{t_{perrow}},$$

where $t_{start}^{\left(i\right)}$ denotes the time that the frame i starting to transfer, and $t_{flash}^{\left(i\right)}$ the time the strobe turns on during the readout phase of the frame i, which results in the smear dot.

**Figure 10:**
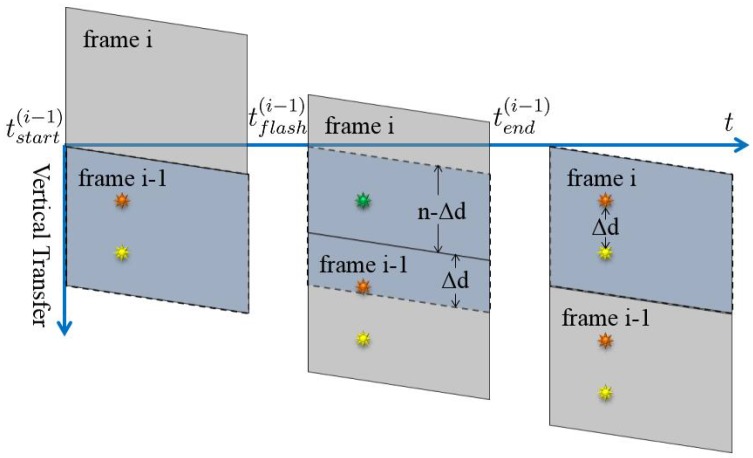
The generation process of the smear dot below the light source position.

If the smear dot appears below the strobe position, shown in Figure 10, the smear is generated during the readout phase of the last frame:

(6) $$\Delta d^{\left(i\right)}=n-\frac{t_{flash}^{\left(i-1\right)}-t_{start}^{\left(i-1\right)}}{t_{perrow}}.$$

For the frame alignment of multiple video cameras, we actually need the $t_{start}^{\left(i\right)}$ for all cameras to be the same. From Equations (5) and (6), we can see that $t_{start}^{\left(i\right)}$ is determined by $\Delta d^{\left(i\right)}$, $t_{flash}^{\left(i\right)}$, $t_{perrow}$ and *n*. We make the cameras capture the same strobe light, so $t_{flash}^{\left(i\right)}$ is the same. Inexpensive cameras in the same model still have good accuracy and stability with respect to frame rate, so $t_{perrow}$ stays consistent. To capture videos with the same resolution m×n, *n* is the same. Therefore, to get the same $t_{start}^{\left(i\right)}$, we only need to adjust the $\Delta d^{\left(i\right)}$ to be the same for all of the cameras.

The smear dot is actually utilized as the time stamp. More specifically, the frame alignment is done by simply adjusting the relative position between the stroboscope and the smear dot, which could be controlled by resetting the shutter. For Canon PowerShot G12, the relative position would be displayed on the preview screen, and the start time of the shutter could be adjusted using the button for switching between different resolutions in the video mode. Through the experiments, the smear dots would appear at the expected position within five trials.

Drawing a conclusion, the frame alignment can be realized by the following settings:

- Set the flash rate of the strobe to the same value as the frame rate of cameras;
- Keep the only smear dot on the same side of the light source for all camera images;
- Adjust the smear dot positions to make them equidistant from the light source.

### 2.5. Sequence Match

**Figure 11:**
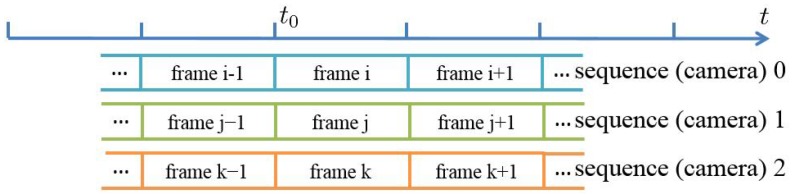
Sequence match. The frames from three video sequences are aligned. The sequence match process is to determine the values of i, j and k.

Given frame aligned video sequences, to realize the synchronization, we should determine the offset time among the sequences. As shown in Figure 11, the frames from three video sequences are aligned, and then the exact values of i, j and k must be obtained to realize the synchronization. We define this process as the sequence match.

**Figure 12:**
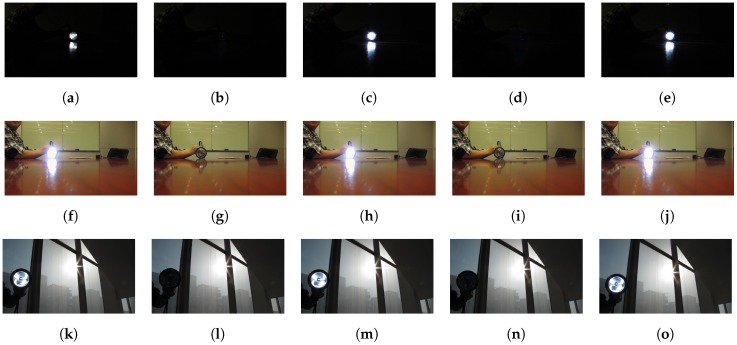
Continuous frames captured from different scenes for flash detection. The frames of the top row (**a**–**e**), the middle row (**f**–**j**) and the bottom row (**k**–**o**) are captured in the environments under weak, medium and strong illumination separately.

To present obvious and stable signals that could be easily and robustly detected, we use the stroboscope to generate periodic flashes with a rate of half of the video frame rate. By controlling the start time of stroboscope, the flashes could be easily caught by the frame aligned cameras. To demonstrate the availability of our approach, we capture the flash sequences in the environments under different strengths of illumination. As shown in Figure 12, for each video sequence, the flash frames are well captured with one interval frame without flash.

The frames from one video sequence could be divided into two parts: odd-index part and even-index part, and the flash frames could be either in an odd-index part or in an even-index part. In the following, we refer to the odd-index or even-index part, which contain the flash frames as a flash subsequence for convenience. In order to realize the sequence match, we design the following feature for each frame:

(7) $$O=\sum_{x,y}C\left(x,y\right),$$

(8) I(x,y)=R(x,y)+G(x,y)+B(x,y),

(9) $$C\left(x,y\right)=\left\{\begin{array}{cc} 1, & I(x,y)>T,\\ 0, & otherwise, \end{array}\right.$$

where R(x,y), G(x,y) and B(x,y) (ranged from 0 to 255) denote the RGB (red, green, blue) values of pixel (x,y) separately and C(x,y) is an indicator function. When the sum of RGB values I(x,y) (ranged from 0 to 765) is larger than the threshold *T*, we set C(x,y) to 1, otherwise, we set C(x,y) to 0. Therefore, for each frame, *O* denotes the number of pixels, of which the sum of RGB values are larger than *T*.

Under different capture circumstances, a const threshold *T* may not work well, so we present an adaptive method to find the threshold automatically. Given a video sequence, we first calculate I(x,y) for each pixel in the sequence. According to the fact that the flash sequence is either in the odd-index part or in the even-index part. Then, for each part, we divide the range [0, 765] into 51 bins, respectively. An appropriate number of bins is important to show the statistic property, and choosing a too large or too small value will lead to poor results. In addition, to divide the range evenly, we set the number of bins to 51 by trial and error. For each bin, we count the number of pixels of which the values I(x,y) fall in the range:

(10) $$R^{\left(i,j\right)}=\left|N\left(I_{odd}^{\left(i,j\right)}\right)-N\left(I_{even}^{\left(i,j\right)}\right)\right|,$$

where $N\left(I_{odd}^{\left(i,j\right)}\right)$ and $N\left(I_{even}^{\left(i,j\right)}\right)$ are the number of pixels that in the bin ranged from *i* to *j* of the odd-index and even-index frames, respectively.

**Figure 13:**
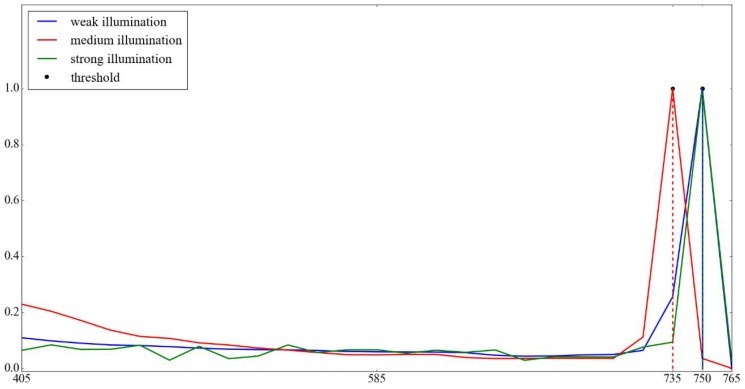
The determination of the threshold. Axis *x* denotes the value of *I* and axis *y* denotes the normalized difference value between the odd-index and even-index frames. The curves shows the normalized differences between the odd-index and even-index frames for each bin, captured under different illumination circumstances in the range from 405 to 765. The start number of the bin with the largest difference is chosen as the threshold.

Figure 13 shows the normalized differences for the video sequences as shown in Figure 12. Considering the two-frame periodic matching signals, the strobe light appears in one frame and disappears in the next frame. In addition, the video sequences are captured successively, so the contents in two adjacent frames would not change too much, except for the periodic flashes of the stroboscope. Furthermore, the values of I(x,y) for the strobe light pixels are always larger than 405 through observations. Therefore, the value I(x,y) of the strobe light should be larger than 405 and in the bin with the largest difference, and we choose the start number of the bin that contains the strobe light pixels as the threshold *T*, which could be described as:

(11) $$T=\underset{i}{\mathrm{argmax}}R^{\left(i,j\right)},i>405.$$

**Figure 14:**
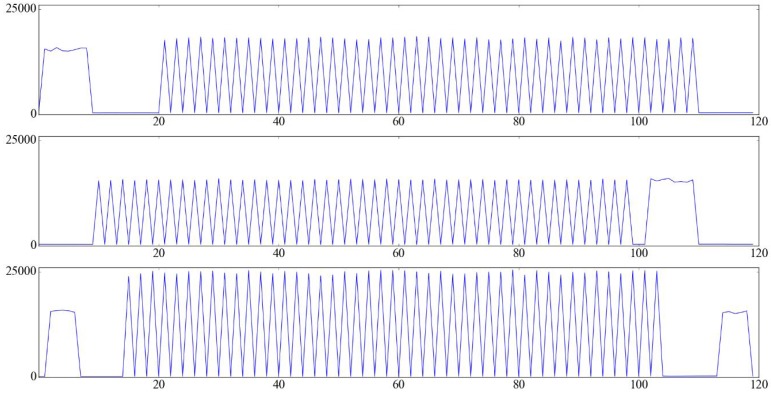
Visualization of feature *O* from multiple video sequences captured under medium illumination. Axis *x* denotes the frame index and axis *y* denotes the value of *O*.

After the calculation of *T*, we could get the *O* values using the Equations (7)–(9). Figure 14 shows the *O* values of frames from multiple videos captured under medium illumination. To determine whether the odd-index or even-index part of a video sequence is the flash subsequence, we just need to calculate the mean of *O* in each part, respectively, and the part with a larger mean value contains flash frames.

**Figure 15:**
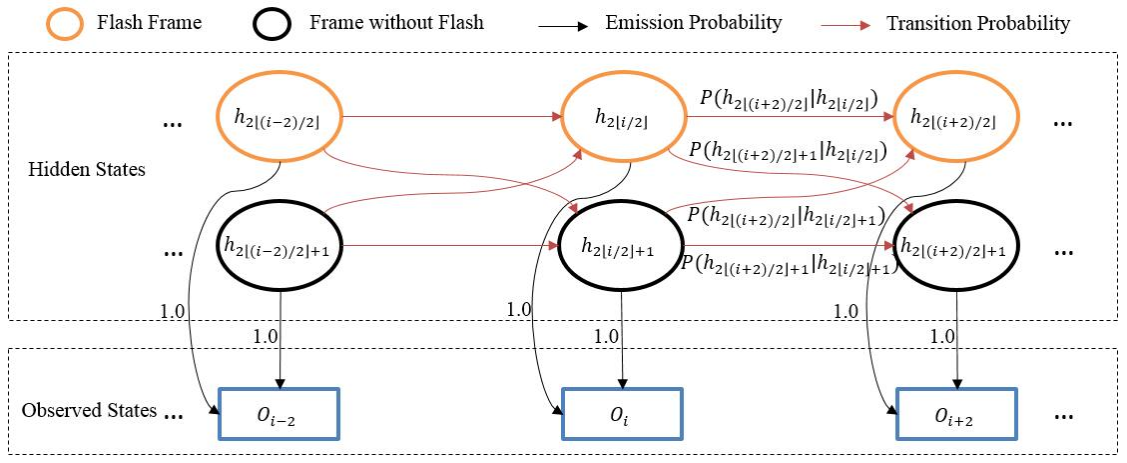
Hidden Markov Model for matching sequences, and the *O* values of the flash subsequences.

After finding the flash subsequences, we apply a hidden Markov model [28] to match the whole sequences. In a hidden Markov model, the input is a sequential series of observed states and the goal is to infer the corresponding sequence of unobserved (hidden) states that is most likely to have generated these observations. Shown in Figure 15, we define the values of *O* of each frame in the flash subsequences as observed states. For each observed state, two hidden states are defined, one hidden state represents that this frame (flash frame) is captured when stroboscope turns on, and the other hidden state represents that this frame (frame without flash) is captured when the stroboscope turns off.

Hidden Markov models require emission probabilities and transition probabilities. The emission probabilities represent the likelihood that a given hidden state will produce a given output. For each frame in the flash subsequences, we define two hidden states and one observed state, so we set the emission probabilities from these two hidden states to the corresponding observed state to 1, and set the emission probabilities from these two hidden states to other observed states to 0. The transition probabilities represent the likelihood of a transition from one hidden state to another hidden state. Through observing patterns of values of *O* in Figure 14, the *O* value of a flash frame is large, followed by a small value in the next frame, which is a frame without flash, and then followed by a large value again, which corresponds to the next flash frame. We find that this large-small-large pattern only occurs when the corresponding frame is a flash frame (except for the last flash frame), and never happens when the frame is a frame without flash. Thus, we draw the conclusion that when the large-small-large pattern occurs, the frame is more likely to be a flash frame. To encourage such a pattern, for each hidden state, we define the transition probabilities as follows:

(12) $$\begin{array}{cc} P_{a} & =P\left(h_{2\lfloor (i+2)/2\rfloor }|h_{2\lfloor i/2\rfloor }\right)\\ & =P\left(h_{2\lfloor (i+2)/2\rfloor }|h_{2\lfloor i/2\rfloor +1}\right)\\ & =\left(1-\frac{{\left(O_{max}-O_{i}\right)}^{2}}{{\left(O_{max}-O_{min}\right)}^{2}}\right)\cdot \frac{{\left(O_{max}-O_{i+1}\right)}^{2}}{{\left(O_{max}-O_{min}\right)}^{2}}\cdot \left(1-\frac{{\left(O_{max}-O_{i+2}\right)}^{2}}{{\left(O_{max}-O_{min}\right)}^{2}}\right), \end{array}$$

(13) $$P_{b}=P\left(h_{2\lfloor (i+2)/2\rfloor +1}|h_{2\lfloor i/2\rfloor }\right)=P\left(h_{2\lfloor (i+2)/2\rfloor +1}|h_{2\lfloor i/2\rfloor +1}\right)=1-P_{a},$$

where $O_{min}$ and $O_{max}$ denotes minimum and maximum value of *O* of all frames from the video sequence. When the large-small-large pattern of the *O* values occurs, $P_{a}$ is close to 1, which means that the current frame is more likely to be a flash frame. Otherwise, $P_{b}$ is close to 1, which means that the current frame is more likely to be a frame without flash. After defining these above probabilities, we can solve this hidden Markov model problem by the Viterbi algorithm [28]. As for the last flash frame, it doesn’t obey the large-small-large pattern, as shown in Figure 14. However, the issue could be easily handled by setting the second frame after the last detected flash frame by the above algorithm as the last flash frame.

For one video sequence, once the hidden Markov model is solved, we get the predicted hidden states for the flash subsequences. Some of these hidden states are predicted as flash frames, and the first flash frame is marked to determine the offset from different video sequences. After applying the above processes to all video sequences, all of the first flash frames are detected, and, as a consequence, the number of offset frames could be utilized to complete the sequence match.

Drawing a conclusion, the sequence match could be realized by the following steps:

- Compute the adaptive threshold *T* based on the video contents,
- Calculate the values of *O* for each frame,
- Get the flash subsequences by choosing the odd-index or even-index subsequence with a larger mean value of *O*,
- Apply the hidden Markov model on the flash subsequences to find the first flash frame, which would be used to determine the offset for each sequence.

## 3. Results

**Figure 16:**
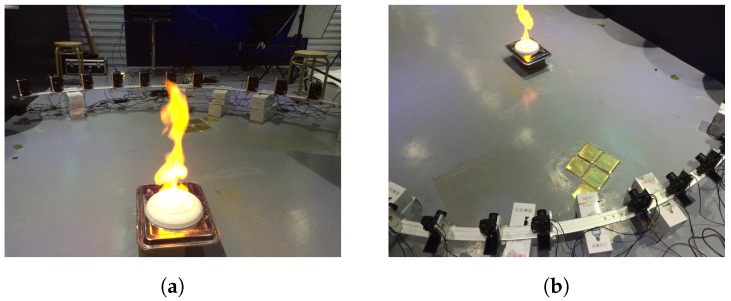
Flame video capture scene. (**a**) One view of the capture scene, (**b**) another view of the capture scene.

The Canon PowerShot G12 cameras are used to capture flames videos with 1280×720 resolution at 23.976 fps frame rate. Figure 16 shows the scene that flame videos are captured with ten G12 cameras. The flash rate of Monarch Instrument Nova-Strobe dbx is set to 23.98 flashes per second in the frame alignment process, and set to 11.99 flashes per second in the sequence match process.

The frame alignment error could be measured by the distance between the strobe and smear dot positions in the image. The resultant time to transfer one row of the pixels is about 54 μs using Equation (4). For videos with 1280×720 resolution, which we use in the experiments, it takes around 720×54μs=38.88 ms to read out the whole image pixels, and the period time for one frame $t_{period}=1s÷23.976\approx 41.7$ ms. Since the adjustment of our synchronization method requires manual intervention, we do not expect to obtain the exact same distances for each camera. However, we can easily set a distance within a 100-pixel offset within five trials for each camera. Therefore, we could easily control the accuracy of our synchronization within 54μs×100=5.4 ms, much less than the frame-level (41.7 ms) synchronization [24,25]. We can achieve more accurate synchronization if the 100-pixel offset distance for each camera is reduced even further with more trials.

**Figure 17:**
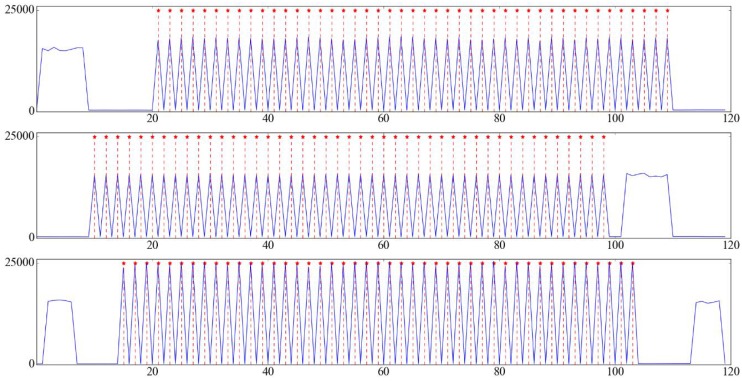
Sequence match result of Figure 14. Axis *x* denotes the frame index and axis *y* denotes the value of *O*. The red star symbols indicate the frames are flash frames.

To evaluate our sequence alignment approach, the periodic flashes videos under different light conditions are captured, as shown in Figure 12. In addition, we also add some noise in the videos to test the robustness of our approach. For example, we move some objects in the scene while capturing the sequences. Figure 17 shows the corresponding results of Figure 14, and we could see that the flash frames are well detected.

We apply our method on 260 captured video sequences, and the start frame of the periodic flashes are all well detected (100%) compared with manually annotated results, which is better than the 85% detection accuracy of the still camera flash based method [24], as shown in Table 1.

**Figure 18:**
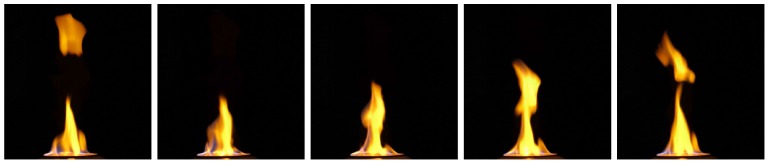
Consecutive flame frames from one camera.

**Figure 19:**
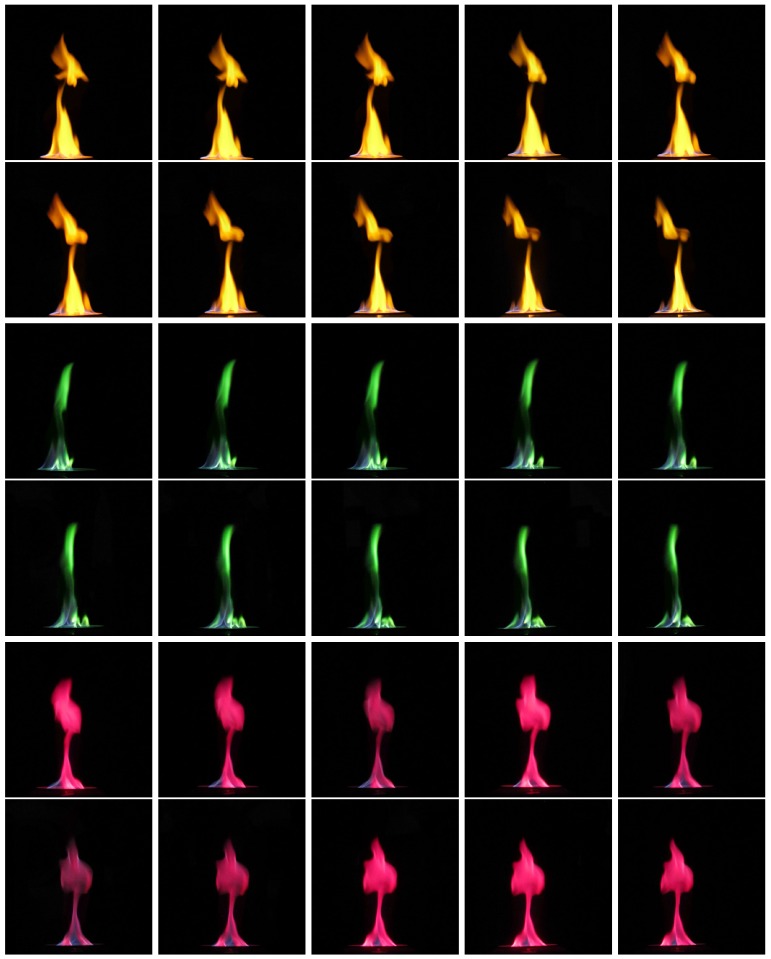
Simultaneously captured flame images from different cameras. Every two rows show the results of one experiment.

With our synchronization approach, we capture flame videos to show the synchronization results. Figure 18 shows a consecutive sequence of five frames from one camera and flames differ greatly even between consecutive frames due to their violent motion. Therefore, if the synchronization accuracy is not good enough, the flame images taken from different views will appear to be totally different, just like those taken from totally different times. Figure 19 shows some results of our synchronization approach.

## 4. Conclusions

In this paper, processes of the imaging and smear generation of full frame CCD sensors are presented, and, based on the numerical analysis of the strobe smear dot, we present a stroboscope based synchronization approach for full frame CCD cameras. To synchronize the video sequences of multiple CCD cameras, we first align the frames from different sequences by adjusting the smear dot positions equidistant from the strobe positions for each camera and then match the flashes to determine the offset time among cameras using a hidden Markov model. The experiments demonstrate the efficacy and effectiveness of our approach. Utilizing inexpensive CCD consumer cameras and one stroboscope, the presented technique greatly reduces the cost of the demand for the synchronized capture, compared with the high-end industry equipment solutions. Theoretically, the same approach could also be applied on the frame transfer CCD sensors besides the full frame ones.

The limitation of our current approach is that the frame alignment process needs manual smear adjustment. However, after only a few manual attempts, the approach performs well for the synchronization of CCD cameras. In addition, if a certain electrical reset method of the shutter could be proposed for the consumer CCD cameras, just like the Casio EX-100Pro could be controlled by an Android app, based on our approach, the synchronization could be done automatically with some image processing methods to detect the positions of the stroboscope and the smear dot. The automatic synchronization process would be similar to the autofocus function for current cameras.

Since the accuracy of the frame alignment is influenced by the smear position and we currently manually adjust the position by trial and error, in the future, we would like to explore a more efficient and elegant way to control the smear position and measure distance from the center of light source to the smear precisely.

## Figures and Tables

**Table 1:** Results of synchronization signal method.

	Method	Still Camera Flash Based Method [24]	Our Method
Result			
Manually annotated		238	260
Correctly detected		210 (88.2%)	260 (100%)
Falsely detected		0 (0%)	0 (0%)
Missed detected		28 (11.8%)	0 (0%)
